# Adjudicated Morbidity and Mortality Outcomes by Age among Individuals with HIV Infection on Suppressive Antiretroviral Therapy

**DOI:** 10.1371/journal.pone.0095061

**Published:** 2014-04-11

**Authors:** Christopher J. Miller, Jason V. Baker, Alison M. Bormann, Kristine M. Erlandson, Katherine Huppler Hullsiek, Amy C. Justice, Jacqueline Neuhaus, Roger Paredes, Kathy Petoumenos, Deborah Wentworth, Alan Winston, Julian Wolfson, James D. Neaton, for the INSIGHT SMART and ESPRIT Study Groups

**Affiliations:** 1 Division of Biostatistics, School of Public Health, University of Minnesota, Minneapolis, Minnesota, United States of America; 2 Division of Infectious Diseases, University of Minnesota, Minneapolis, Minnesota, United States of America; 3 Hennepin County Medical Center, Minneapolis, Minnesota, United States of America; 4 Division of Infectious Diseases, University of Colorado Anschutz Medical Campus, Aurora, Colorado, United States of America; 5 Veterans Affairs Connecticut Healthcare System, West Haven, Connecticut, United States of America; 6 School of Medicine and Public Health, Yale University, New Haven, Connecticut, United States of America; 7 irsiCaixa Foundation, Hospital Universitari Germans Trias i Pujol, Universitat Autónoma de Barcelona, Badalona, Spain; 8 Lluita contra la SIDA Foundation, Badalona, Spain; 9 The Kirby Institute, University of New South Wales, Sydney, Australia; 10 Department of Medicine, Imperial College, London, England; Rush University, United States of America

## Abstract

**Background:**

Non-AIDS conditions such as cardiovascular disease and non-AIDS defining cancers dominate causes of morbidity and mortality among persons with HIV on suppressive combination antiretroviral therapy. Accurate estimates of disease incidence and of risk factors for these conditions are important in planning preventative efforts.

**Methods:**

With use of medical records, serious non-AIDS events, AIDS events, and causes of death were adjudicated using pre-specified criteria by an Endpoint Review Committee in two large international trials. Rates of serious non-AIDS which include cardiovascular disease, end-stage renal disease, decompensated liver disease, and non-AIDS cancer, and other serious (grade 4) adverse events were determined, overall and by age, over a median follow-up of 4.3 years for 3,570 participants with CD4^+^ cell count ≥300 cells/mm^3^ who were taking antiretroviral therapy and had an HIV RNA level ≤500 copies/mL. Cox models were used to examine the effect of age and other baseline factors on risk of a composite outcome of all-cause mortality, AIDS, or serious non-AIDS.

**Results:**

Five-year Kaplan-Meier estimates of the composite outcome, overall and by age were 8.3% (overall), 3.6% (<40), 8.7% (40–49) and 16.1% (≥50), respectively (p<0.001). In addition to age, smoking and higher levels of interleukin-6 and D-dimer were significant predictors of the composite outcome. The composite outcome was dominated by serious non-AIDS events (overall 65% of 277 participants with a composite event). Most serious non-AIDS events were due to cardiovascular disease and non-AIDS cancers.

**Conclusions:**

To date, few large studies have carefully collected data on serious non-AIDS outcomes. Thus, reliable estimates of event rates are scarce. Data cited here, from a geographically diverse cohort, will be useful for planning studies of interventions aimed at reducing rates of serious non-AIDS events among people with HIV.

## Introduction

Potent combination antiretroviral therapy (cART) has improved life expectancy for people with HIV. AIDS-related events are now less common among patients taking suppressive cART [1]. Instead, morbidity and mortality is dominated by serious non-AIDS (SNA) conditions, particularly cardiovascular disease (CVD), end-stage renal disease, decompensated liver disease and non-AIDS defining cancer. Studies comparing persons with and without HIV infection have shown that HIV-positive individuals have higher rates of heart failure [2]_ENREF_12, myocardial infarction [3]–[5], stroke [6], and cancer [7]–[9]. A recent review considers several possible reasons for the excess risk of SNA events among HIV positive individuals [10]. These reasons include cART, traditional risk factors, and immune dysfunction and inflammation. Possible therapeutic approaches are discussed in the review.

Future intervention trials will require accurate estimates of SNA event rates and of patient risk factors that could be used to select study participants. In this report, we take advantage of the long-term follow-up and centrally adjudicated clinical outcomes of participants in the control arms of two large international randomized clinical trials who received continuous cART aimed at viral suppression to estimate rates of a composite outcome of all-cause mortality, SNA, or AIDS, and rates for each component of this composite outcome. Results are given overall and by age since risk of SNA increase with age and, therefore, age is an obvious factor to consider as an inclusion criterion in future trials. Furthermore, the number of people aged ≥50 years living with HIV-1 (HIV) has been increasing worldwide [11]–[13], and this motivates the study of novel interventions to prevent SNA diseases.

## Methods

### Study Population

Outcomes for the participants in the control arms of the Strategies for Management of Anti-Retroviral Therapy (SMART) trial and the Evaluation of Subcutaneous Proleukin in a Randomized International Trial (ESPRIT) are the subject of this report. Both studies were carried out by the International Network for Strategic Initiatives in Global HIV Trials (INSIGHT). The study design and methods of both studies have been reported previously [14]–[16]. In SMART, 5472 HIV-infected individuals with CD4^+^ cell counts >350 cells/mm^3^ were randomized to either the Drug Conservation group, which received CD4^+^ cell count-guided episodic cART or the Viral Suppression control group, which received continuous cART [16]. Enrollment in SMART ended in 2006 and all participants were followed through July 2007 [15]. In ESPRIT, 4111 HIV-infected individuals with CD4^+^ cell counts ≥300 cells/mm^3^ were randomized to receive cART alone (control group) or cART with interleukin-2 [14]. Enrollment in ESPRIT ended in 2003 and all participants were followed through November 2008.

For this study, we included participants in the control arms of SMART and ESPRIT who were on cART at study entry with an HIV RNA level ≤500 copies/mL (a lower limit of detection that could be applied at all sites for both studies) to focus on participants who were being successfully treated with cART. This now represents the great majority of patients taking ART and is likely to be the target population for adjunctive interventions aimed at reducing SNA since the first goal of treatment is maximal and durable suppression of plasma HIV viral load [17]. Neither SMART nor ESPRIT specified cART regimens to be used to maintain suppressed HIV RNA levels.

Written informed consent was obtained from all participants. The institutional review board (IRB) or institutional ethics committee (IEC) at each site and the University of Minnesota, which served as the Statistical and Data Management Center, approved the protocols of SMART and ESPRIT and the analysis of stored specimens for consenting participants. The University of Minnesota institutional review board also approved plans for the continued analysis of SMART and ESPRIT datasets utilized in this study. Copies of all IRB/IEC approval letters are filed with the Statistical and Data Management Center at the University of Minnesota.

### AIDS and Non-AIDS Clinical Outcomes and Causes of Death

The INSIGHT Endpoint Review Committee (ERC) reviewed AIDS and SNA non-fatal events and deaths using pre-specified criteria [18], [19]. Briefly, non-fatal event documentation was reviewed by three reviewers and differences were adjudicated. Events considered “confirmed” or “probable” based on the pre-specified criteria were counted endpoints. Causes of death were classified by the ERC using the CoDe system [20], [21].

Serious non-AIDS (SNA) events included CVD, end-stage renal disease, decompensated liver disease, and non-AIDS cancer (excluding basal and squamous cell skin cancers). Myocardial infarction, cerebrovascular strokes, coronary artery disease requiring an invasive procedure, and CVD death were classified as CVD events.

In addition to AIDS and SNA events, rates of grade 4 events and estimated glomerular filtration (eGFR) are also reported. Grade 4 events are potentially life-threatening symptomatic events requiring medical intervention according to the toxicity table of the Division of AIDS of the National Institute of Allergy and Infectious Diseases (NIAID). In each study grade 4 events were classified using System Organ Classes (SOC) defined by the Medical Dictionary for Regulatory Activities (MedDRA). eGFR was calculated using the Chronic Kidney Disease-Epidemiology Collaboration equation [22]. Chronic kidney disease (CKD) was defined as a 25% decline in eGFR to a value <60 mL/min/1.73 m^2^ from study entry.

### Baseline and Follow-up Visit Data Collection

Medical and treatment histories, including AIDS and non-AIDS diagnoses, HIV and SNA risk factors, and use of concomitant medications, were ascertained prior to randomization. Interleukin-6 (IL-6), an inflammatory marker, and D-dimer, a coagulation marker, were measured at baseline on stored specimens in both studies for consenting participants [23], [24].

In ESPRIT, follow-up visits for data collection were conducted every four months; in SMART, participants were seen one month and two months after enrollment, then in two-month intervals until year one, and in four-month intervals in subsequent years. CD4^+^ cell count and HIV RNA levels were measured locally at each follow-up visit. For consenting individuals, stored specimens were used to measure creatinine levels annually with additional measurements at four and eight months in SMART.

### Statistical analysis

Participant characteristics at enrollment and outcomes during follow-up are presented overall and for three age groups: <40, 40–49, and ≥50 years of age. Cross-sectional associations of demographic and health factors with age were assessed using logistic and general linear models for categorical and continuous variables, respectively. Event rates per 100 person-years are cited and cumulative three, four and five-year event probabilities are calculated using the Kaplan-Meier method with 95% log-log confidence intervals (CIs). Cox proportional hazards models were used to examine the relationship between age and major clinical outcomes, and to investigate determinants of a composite outcome of all-cause mortality, SNA, or AIDS; adjusted hazard ratios (aHRs) for ten years increase in age and 95% CIs are cited. For each event considered, time at risk was defined as the time from randomization to the first event, death, loss to follow-up, or study closure.

Data were analyzed using SAS version 9.3 and R version 2.15.1. *P*-values are two-sided and unadjusted for multiple comparisons.

## Results

### Study participants

Among 4792 participants in the control arms of SMART (n = 2752) and ESPRIT (n = 2040), 3570 (74.5%) were taking cART and had an HIV RNA level ≤500 copies/mL at study entry. Of these participants from 35 countries, 1327 (37.2%) were enrolled by sites in Europe; 1319 (36.9%) by sites in North America; 489 (13.7%) by sites in South America; 257 (7.2%) by sites in Asia; 146 (4.1%) by sites in Australia; and 32 (0.9%) by sites in Africa. Median follow-up time was 4.3 years (interquartile range [IQR]: 2.1 and 6.7 years). Vital status was known for 96.5% of participants at the completion of both studies.

Table 1 summarizes baseline characteristics. Median age was 42 years (IQR: 36–49); 1361 (38%), 1379 (39%), and 830 (23%) participants were aged <40 years, 40–49 years, and ≥50 years, respectively. Older participants were more likely to be male (*P*<0.001) and had acquired HIV through homosexual contact (*P*<0.001). As expected, older participants were more likely to have had a history of CVD or diabetes (both *P*<0.001), and were more likely to be on medications for hypertension, hyperlipidemia, osteoporosis, diabetes, or CVD (all *P*<0.001). Among participants ≥50 years old, 14.6% were taking two or more of these types of medication in addition to cART. Overall, the median time on ART was 4.0 years (IQR: 2.6–5.0). Older participants were more likely to have been on ART longer and have a history of use of protease inhibitors (PIs) and non-nucleoside reverse transcriptase inhibitors (NNRTIs). Older participants also were more likely to have been prescribed abacavir, tenofovir, stavudine, zalicitabine, and lamivudine.

**Table 1:** Participant characteristics at study entry overall by age category.

Characteristic	Overall	<40 years	40–49 years	≥50 years	P-value
N (ESPRIT, SMART)	3570 (1636, 1934)	1361 (779, 582)	1379 (556, 823)	830 (301, 529)	
Demographics					
Female gender	815 (22.8%)	397 (29.2%)	284 (20.6%)	134 (16.1%)	<0.001
Black race	628 (17.6%)	212 (15.6%)	270 (19.6%)	146 (17.6%)	0.35
Likely modes of transmission					
Homosexual	1861 (52.1%)	656 (48.2%)	737 (53.4%)	468 (56.4%)	<0.001
Heterosexual	1442 (40.4%)	600 (44.1%)	521 (37.8%)	321 (38.7%)	<0.001
Intravenous drug use	335 (9.4%)	131 (9.6%)	160 (11.6%0	44 (5.3%)	<0.001
Medical History					
Years diagnosed with HIV	7.3 (4.2, 11.4)	5.6 (3.2, 9.0)	8.3 (5.1, 12.5)	8.9 (5.2, 13.2)	<0.001
CD4^+^ cell count (cells/mm^3^)	547 (421, 949)	528 (415, 686)	562 (432, 758)	557 (420, 730)	0.006
Nadir CD4^+^ (cells/mm^3^)	209 (105, 313)	223 (120, 322)	200 (93, 312)	200 (104, 293)	0.001
Prior AIDS diagnosis	917 (25.7%)	289 (21.2%)	385 (27.9%)	243 (29.3%)	<0.001
Hepatitis B or C infection#	597 (18.2%)	218 (17.8%)	272 (21.1%)	107 (13.9%)	0.04
Body mass index (kg/m^2^)	24.1 (22.0, 26.6)	23.7 (21.6, 26.0)	24.2 (22.1, 26.8)	24.6 (22.6, 27.0)	<0.001
Current smoker#	696 (36.0%)	202 (24.7%)	326 (39.6%)	168 (31.8%)	0.04
History of CVD event	84 (2.4%)	6 (0.4%)	33 (2.4%)	45 (5.4%)	<0.001
Polypharmacy%	235 (6.6%)	23 (1.7%)	91 (6.6%)	121 (14.6%)	<0.001
Diabetes mellitus	173 (4.9%)	16 (1.2%)	74 (5.4%)	83 (10.0%)	<0.001
Lipodystrophy #	565 (29.2%)	112 (19.2%)	259 (31.5%)	194 (36.7%)	<0.001
cART History					
Duration of cART (years)	4.0 (2.6, 5.0)	3.0 (2.0, 5.0)	4.2 (3.0, 5.0)	4.3 (3.0, 5.0)	<0.001
PI use at study entry	1640 (45.9%)	582 (42.8%)	678 (49.2%)	380 (45.8%)	0.06
NNRTI use at study entry	1796 (50.3%)	672 (49.4%)	680 (49.3%)	444 (53.5%)	0.10
Only NRTI use at study entry	387 (10.8%)	171 (12.6%)	136 (9.9%)	80 (9.6%)	0.03
Any PI exposure	2596 (72.7%)	902 (66.3%)	1047 (75.9%)	647 (78.0%)	<0.001
Any NNRTI exposure	2303 (64.5%)	840 (61.7%)	904 (65.6%)	559 (67.4%)	0.002
Abacavir use at study entry	794 (22.2%)	254 (18.7%)	332 (24.1%)	208 (25.1%)	<0.001
Tenofovir use at study entry	435 (12.2%)	124 (9.1%)	187 (13.6%)	124 (15.0%)	<0.001
Lamivudine (3TC) use at study entry	2782 (77.9%)	1058 (77.7%)	1070 (77.6%)	654 (78.8%)	0.98
Stavudine (d4T) use at study entry	1004 (28.1%)	423 (31.1%)	373 (27.1%)	208 (25.1%)	<0.001
Didanosine (ddI) use at study entry	586 (16.4%)	253 (18.6%)	236 (17.1%)	97 (11.7%)	<0.001
Zalcitabine (ddC) use at study entry	20 (0.6%)	9 (0.7%)	8 (0.6%)	3 (0.4%)	0.33
Zidovudine (AZT) use at study entry	1716 (48.1%)	700 (51.4%)	634 (46.0%)	382 (46.0%)	0.003
Any abacavir exposure	983 (27.5%)	319 (23.4%)	413 (30.0%)	251 (30.2%)	<0.001
Any tenofovir exposure	538 (15.1%)	170 (12.5%)	228 (16.5%)	140 (16.9%)	<0.001
Any lamivudine (3TC) exposure	3342 (93.6%)	1252 (92.0%)	1302 (94.4%)	788 (94.9%)	0.03
Any stavudine (d4T) exposure	2126 (59.6%)	766 (56.3%)	847 (61.4%)	513 (61.8%)	<0.001
Any didanosine (ddI) exposure	1539 (43.1%)	564 (41.4%)	636 (46.2%)	339 (40.8%)	0.33
Any lamivudine (ddC) exposure	521 (14.6%)	150 (11.0%)	236 (17.1%)	135 (16.3%)	<0.001
Any zidovudine (AZT) exposure	3022 (84.6%)	1145 (84.1%)	1172 (85.0%)	705 (84.9%)	0.43
Laboratory values					
eGFR∧ (mL/min/1.73 m^2^)	110 (99, 120)	118 (110, 127)	110 (102, 118)	99 (89, 107)	<0.001
Total:HDL cholesterol#	4.6 (3.6, 6.0)	4.2 (3.3, 5.6)	4.8 (3.7, 6.2)	4.8 (3.8, 6.2)	<0.001
D-dimer* (μg/mL)	0.22 (0.15, 0.35)	0.20 (0.14, 0.31)	0.22 (0.14, 0.34)	0.23 (0.17, 0.45)	<0.001
Interleukin-6* (pg/mL)	1.73 (1.10, 2.74)	1.50 (0.95, 2.36)	1.70 (1.15, 2.69)	2.27 (1.44, 3.40)	<0.001
hsCRP (μg/mL)	1.58 (0.68, 3.67)	1.17 (0.53, 2.98)	1.63 (0.75, 3.80)	2.14 (0.99, 5.11)	<0.001

Values are median (IQR) or n (%). Significance tests for age differences are general linear models or logistic regression models for continuous and categorical variables, respectively, with age analyzed as a continuous variable. #Data unavailable for either all ESPRIT participants or for a subset of ESPRIT participants who enrolled from the Vanguard studies. *Biomarker summary statistics reflect a subset of the sample (n = 1193, 1253, and 775 for age strata, respectively). %Polypharmacy was considered as use of two or more of the following drug classes hypertensive, hyperlipidaemia, coronary artery disease, osteoporosis, or diabetes. ∧eGFR was available for participants who consented to store blood samples (n = 960, 1011, and 640 for age strata, respectively). cART = combination antiretroviral therapy. NRTI = nucleoside analog reverse transcriptase inhibitor. PI = protease inhibitor. NNRTI = non-nucleoside analog reverse transcriptase inhibitor. eGFR = estimated glomerular filtration rate. hsCRP = high-sensitivity c-reactive protein. CVD = cardiovascular disease. History of CVD event includes history of myocardial infarction, coronary artery disease surgery, or stroke.

Total/HDL cholesterol, IL-6, and D-dimer levels were higher and eGFR was lower among older participants (all *P*<0.001).

### Rates of major clinical events

During follow-up, 113 participants died (Table 2). Most common causes of death for aged <40 years that could be adjudicated were CVD (23%), drug overdose (13%), non-AIDS cancer (10%) and AIDS (10%). Causes of death for 19 participants could not be determined. Common causes of death for older persons aged 40–49 years were non-AIDS cancer (28%), liver disease (11%), CVD (8%) and infection (8%). The most common causes of death among participants aged ≥50 years were cancer (28%) and CVD (20%). Overall, only 6% of deaths were AIDS-related; 45% were attributable to causes other than AIDS or SNA (i.e., accident, infection, substances, suicide, or other).

**Table 2:** Morbidity and mortality event rates by age.

Event	Overall		<40 years		40–49 years		≥50 years		aHR (95% CI)	P-value
	n	Rate (SE)	n	Rate (SE)	n	Rate (SE)	n	Rate (SE)		
All-cause mortality	113	0.70 (0.07)	31	0.45 (0.08)	36	0.61 (0.10)	46	1.31 (0.19)	1.8 (1.5–2.2)	<0.001
AIDS (fatal or non-fatal)	54	0.34 (0.05)	14	0.21 (0.06)	27	0.46 (0.09)	13	0.38 (0.10)	1.2 (0.9–1.6)	0.17
SNA event	170	1.08 (0.08)	25	0.37 (0.07)	62	1.08 (0.14)	83	2.51 (0.28)	2.0 (1.7–2.3)	<0.001
Mortality, AIDS, or SNA	268	1.71 (0.10)	55	0.82 (0.11)	101	1.77 (0.18)	112	3.41 (0.32)	1.7 (1.5–2.0)	<0.001
CVD event	79	0.49 (0.06)	12	0.18 (0.05)	26	0.45 (0.09)	41	1.21 (0.19)	2.1 (1.7–2.6)	<0.001
Non-AIDS related cancers	79	0.49 (0.06)	11	0.16 (0.05)	27	0.46 (0.09)	41	1.20 (0.19)	2.0 (1.6–2.4)	<0.001
Chronic kidney disease∧	89	0.99 (0.11)	23	0.63 (0.13)	29	0.89 (0.17)	37	1.82 (0.30)	1.9 (1.5–2.3)	<0.001
Bacterial pneumonia	106	0.67 (0.07)	35	0.52 (0.09)	45	0.79 (0.12)	26	0.77 (0.15)	1.2 (1.0–1.4)	0.14
All-cause hospitalization	817	5.66 (0.20)	278	4.52 (0.27)	291	5.58 (0.33)	248	8.03 (0.51)	1.3 (1.2–1.4)	<0.001
Any grade-4 event	440	2.96 (0.14)	136	2.14 (0.18)	159	2.92 (0.23)	145	4.71 (0.39)	1.5 (1.3–1.6)	<0.001
Hematologic grade 4 event	15	0.09 (0.02)	5	0.07 (0.03)	5	0.08 (0.04)	5	0.14 (0.06)	1.9 (1.1–3.2)	0.02
Gastrointestinal grade 4 event	66	0.41 (0.05)	20	0.30 (0.07)	24	0.41 (0.08)	22	0.64 (0.14)	1.3 (1.0–1.7)	0.04
Hepatobiliary grade 4 event	22	0.14 (0.03)	5	0.07 (0.03)	7	0.12 (0.04)	10	0.29 (0.09)	1.8 (1.2–2.9)	0.008
Nervous system grade 4 event	53	0.33 (0.05)	19	0.28 (0.06)	15	0.26 (0.07)	19	0.55 (0.13)	1.6 (1.2–2.1)	<0.001
Renal and urinary grade 4 event	31	0.19 (0.03)	11	0.16 (0.05)	8	0.14 (0.05)	12	0.35 (0.10)	1.4 (1.0–2.1)	0.07

N represents the number of patients with an event over follow-up. aHR = adjusted hazard ratio. Significance tests and aHR are from Cox models for the continuous effect of a ten year increase in age adjusted for the effects of gender, likely mode of infection, and study (SMART or ESPRIT). The rate is the number of events per 100 person-years of follow-up with only first events considered (i.e., time-to-event). CVD = cardiovascular disease. SNA = Serious non-AIDS. ∧Data available for participants who consented to storing blood sample for future testing (n = 2688). No grade 4 hepatobiliary events occurred among women.

Rates of a composite outcome with components of death, AIDS or SNA increased with age (aHR per 10 years older: 1.7; 95% CI: 1.5–2.0). In each age group, SNA events were the dominant component of this composite outcome. Overall, the most common SNA events were non-AIDS cancers and CVD (79 participants each). The most common cancers were lung cancer (n = 16), prostate cancer (n = 11), anal cancer (n = 8), breast cancer (n = 7), skin cancer (n = 7), and colon cancer (n = 4). For the 79 participants with at least one CVD event, the individual CVD events were acute myocardial infarction (n = 40), coronary artery disease with surgery (n = 21), stroke (n = 10), and other causes (n = 8). Decompensated liver disease (n = 14) and end stage renal disease (n = 4) were much less common.

Rates of all-cause mortality (aHR: 1.8; 95% CI: 1.5–2.2), SNA events (aHR: 2.0; 95% CI: 1.7–2.3), CVD events (aHR: 2.1; 95% CI: 1.7–2.6), non-AIDS cancers (aHR: 2.0; 95% CI: 1.6–2.4), CKD (aHR: 1.9; 95% CI: 1.5–2.3), and all-cause hospitalization (aHR: 1.3; 95% CI: 1.2–1.4), increased with older age as expected (Table 2). AIDS events were rare and were not significantly related to age (aHR: 1.2; 95% CI: 0.9–1.6). Similarly, risk of bacterial pneumonia did not vary significantly by age (aHR: 1.2; 95% CI: 0.9–1.5). Grade 4 events of multiple etiologies including the hematologic (aHR: 1.9; 95% CI: 1.1–3.2), gastrointestinal (aHR: 1.3; 95% CI: 1.0–1.7), and neurological systems (aHR: 1.6; 95% CI: 1.2–2.1), were more likely with older age.

**Figure 1:**
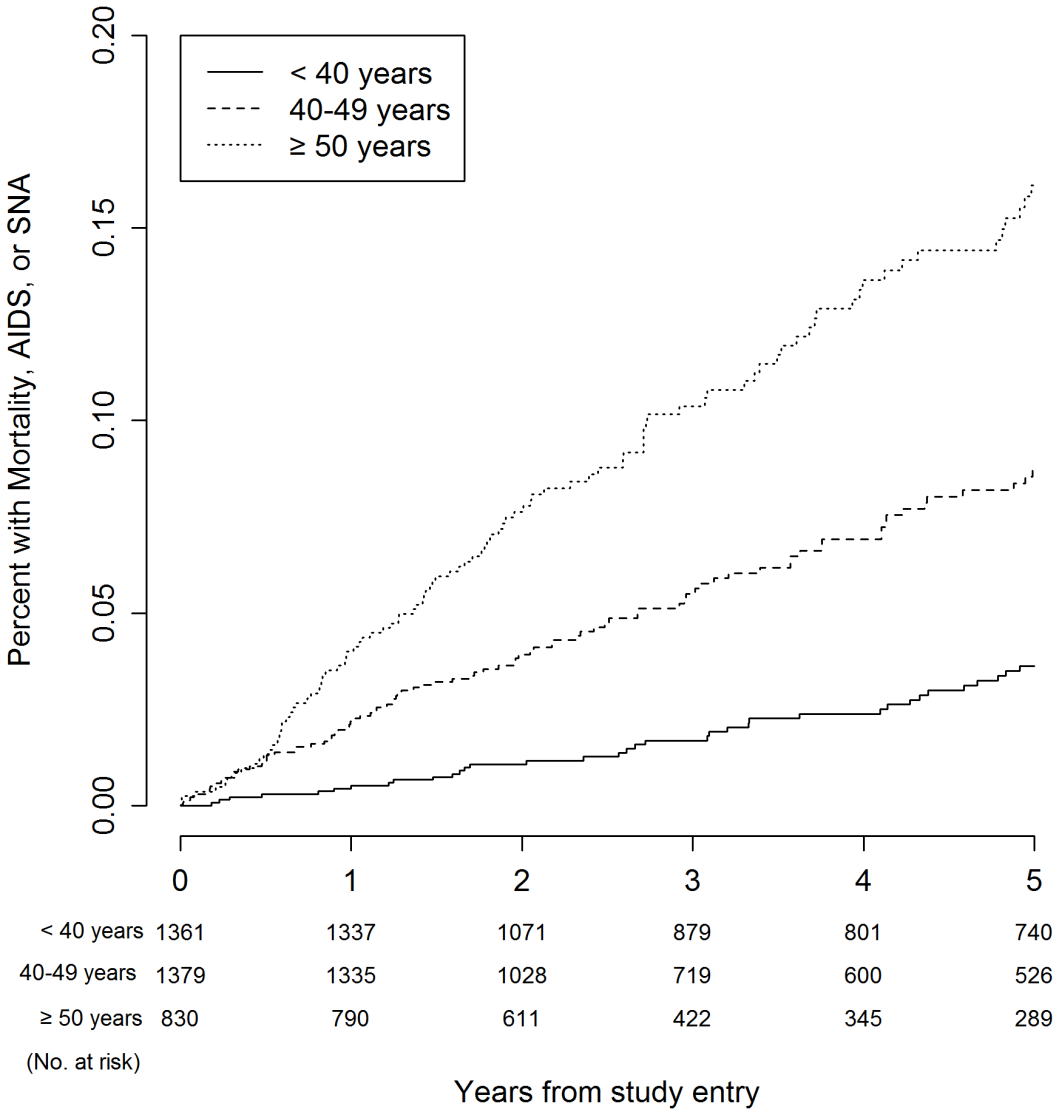
Cumulative probability of composite endpoint (death, SNA, or AIDS) by age group.

**Figure 2:**
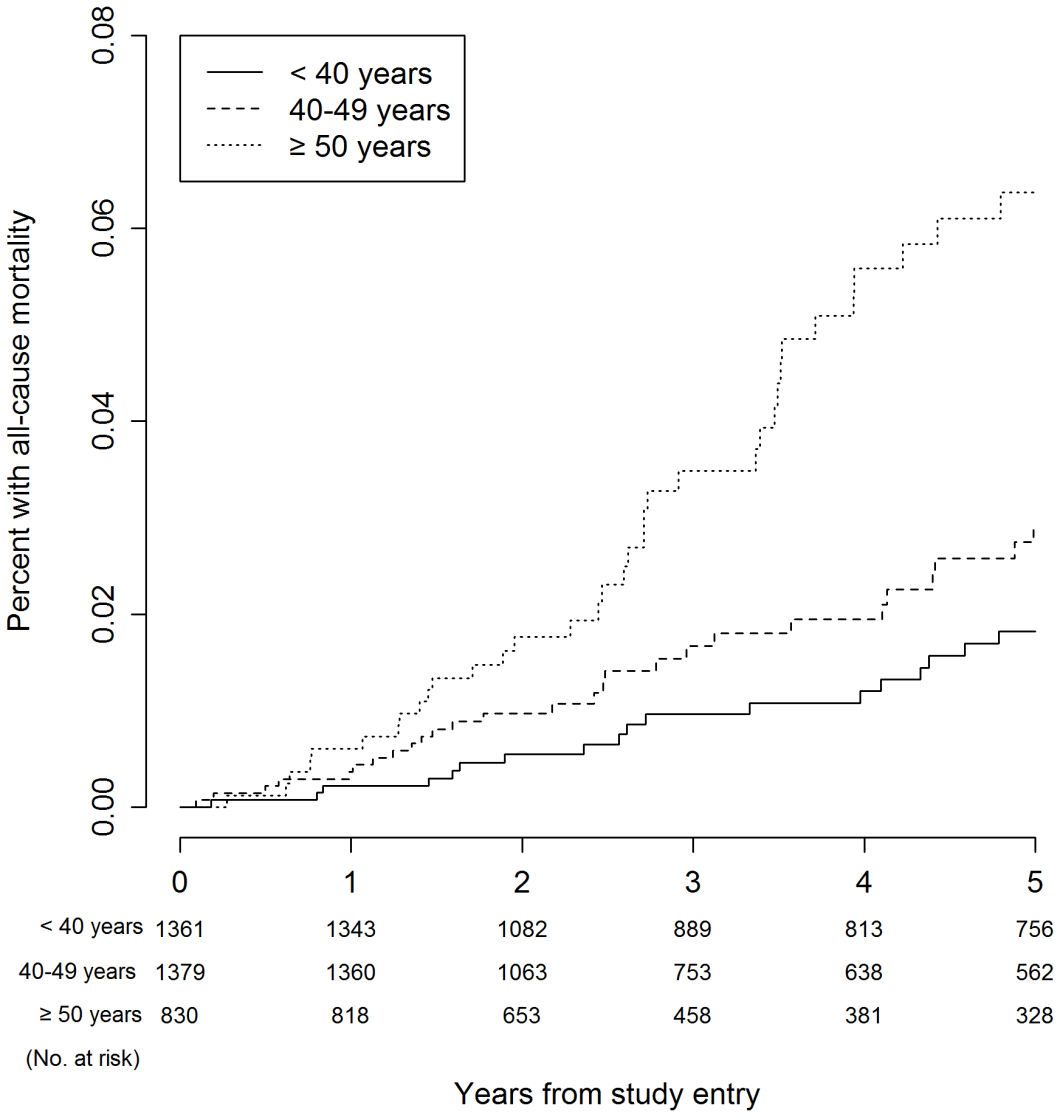
Cumulative probability of death by age group.

**Figure 3:**
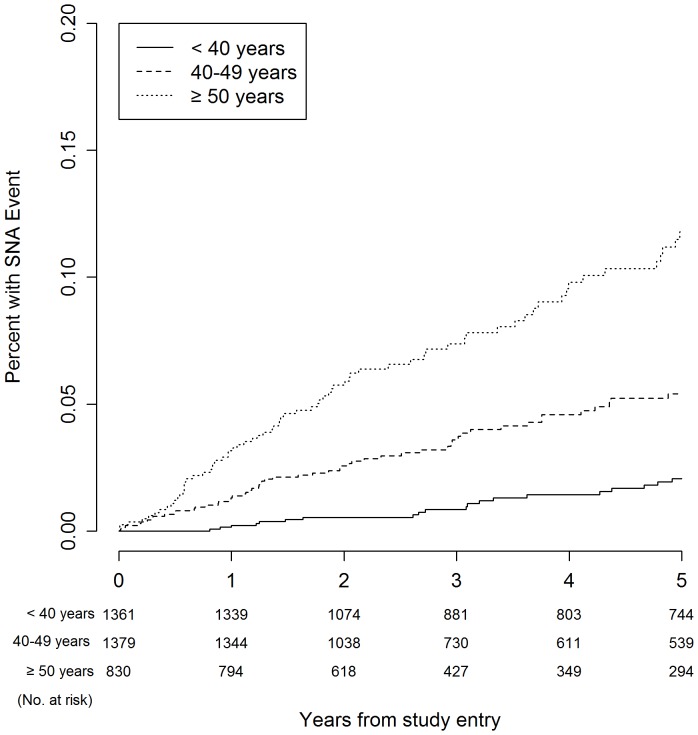
Cumulative probability of SNA event by age group.

**Figure 4:**
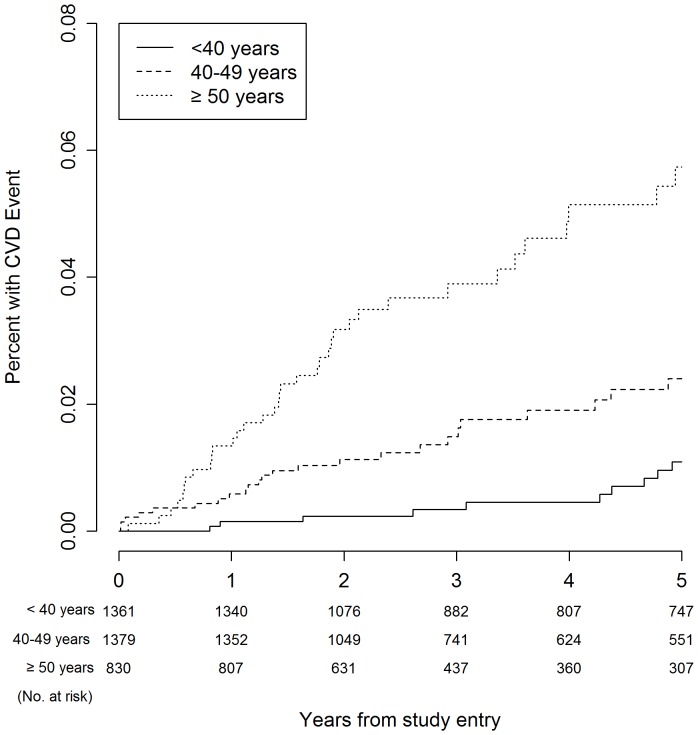
Cumulative probability of CVD event by age group.

**Figure 5:**
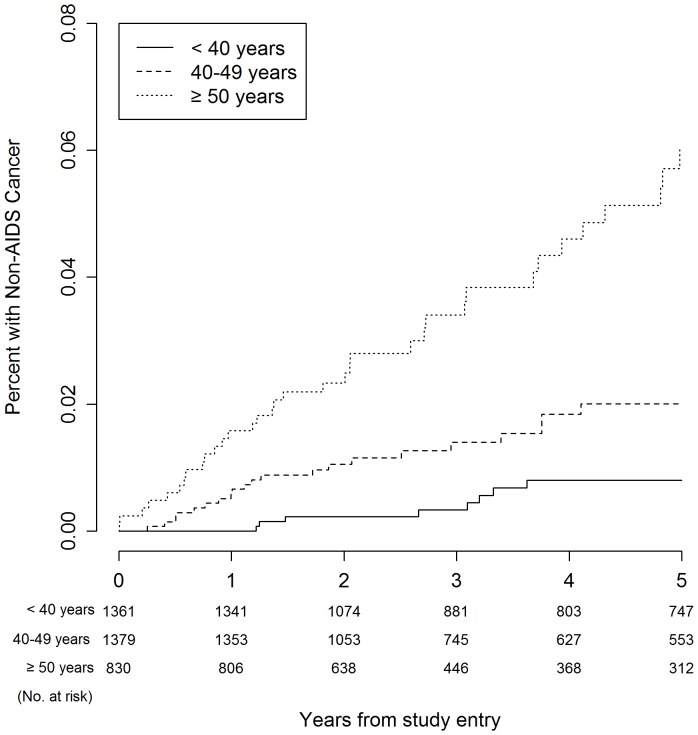
Cumulative probability of non-AIDS cancer by age group.

**Figure 6:**
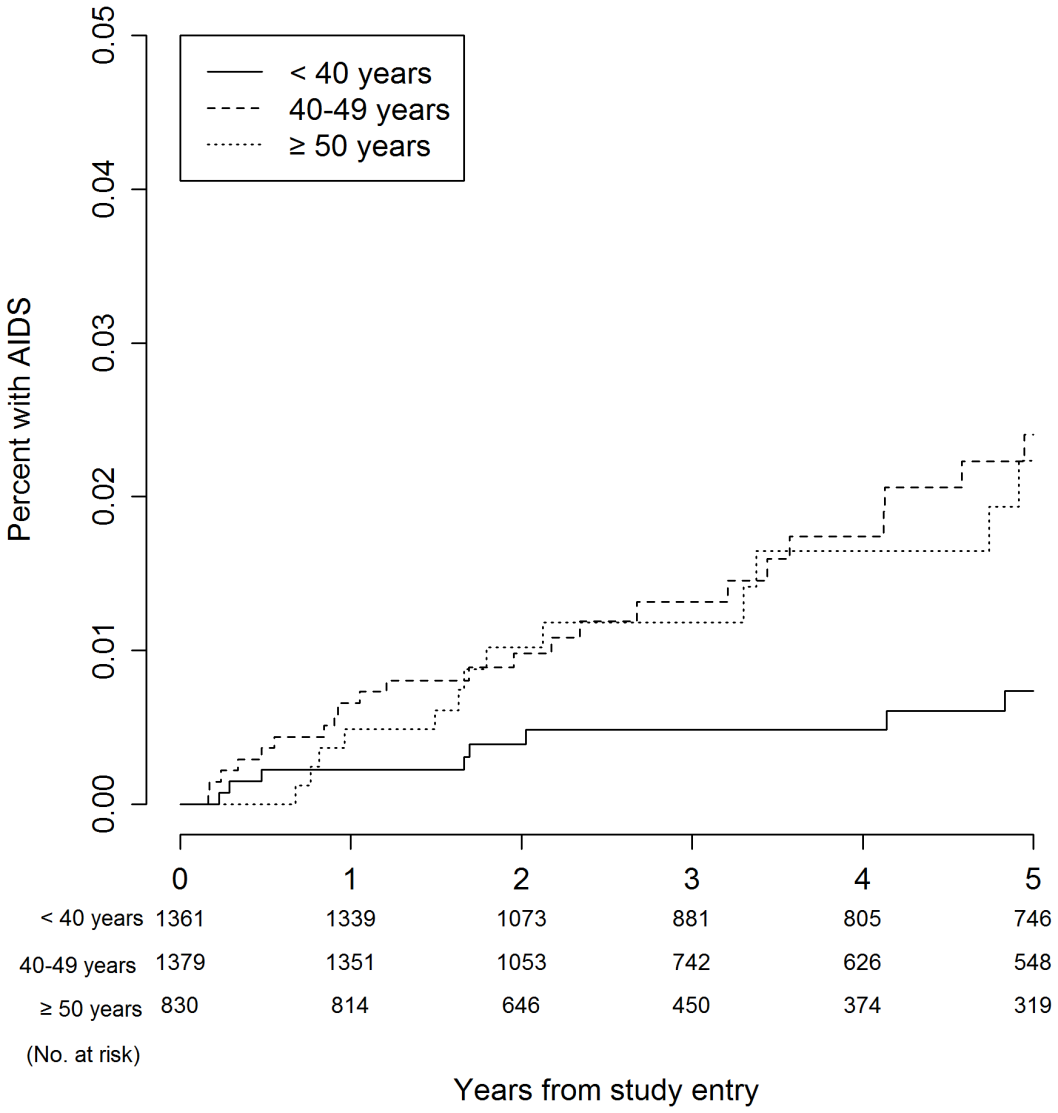
Cumulative probability of AIDS by age group.

Table 3 presents Kaplan-Meier estimates for serious event rates at three, four, and five years. The five-year Kaplan-Meier event rates for all-cause mortality were 1.8%, 2.9%, and 6.4% for participants aged <40 years, 40–49 years, and ≥50 years, respectively (*P*<0.001). With regard to the composite endpoint, five-year event rates were 3.6%, 8.7% and 16.1% for the respective age groups (*P*<0.001). Figures 1–6 show Kaplan-Meier plots for the composite outcome of death, SNA or AIDS and each major component of this composite outcome including CVD and non-AIDS cancer.

**Table 3:** Kaplan-Meier probabilities for events at 3, 4, and 5 years.

Event	Overall	<40 years	40–49 years	≥50 years
	Estimate (95% CI)	Estimate (95% CI)	Estimate (95% CI)	Estimate (95% CI)
All-cause mortality				
3 years	1.8% (1.4–2.4)	1.0% (0.5–1.7)	1.7% (1.1–2.6)	3.5% (2.3–5.3)
4 years	2.5% (1.9–3.2)	1.2% (0.7–2.1)	1.9% (1.3–3.0)	5.6% (3.9–7.9)
5 years	3.2% (2.6–4.1)	1.8% (1.1–2.9)	2.9% (2.0–4.3)	6.4% (4.6–8.9)
AIDS				
3 years	1.0% (0.7–1.4)	0.5% (0.2–1.1)	1.3% (0.8–2.2)	1.2% (0.6–2.3)
4 years	1.2% (0.9–1.7)	0.5% (0.2–1.1)	1.7% (1.1–2.8)	1.6% (0.9–3.0)
5 years	1.7% (1.2–2.3)	0.7% (0.4–1.5)	2.4% (1.6–3.7)	2.2% (1.3–4.0)
CVD event				
3 years	1.6% (1.2–2.1)	0.3% (0.1–0.9)	1.5% (0.9–2.4)	3.9% (2.7–5.6)
4 years	2.1% (1.6–2.7)	0.5% (0.2–1.1)	1.9% (1.2–3.0)	5.1% (3.6–7.2)
5 years	2.6% (2.1–3.4)	1.1% (0.6–2.0)	2.4% (1.6–3.7)	5.7% (4.1–8.0)
Non-AIDS cancer				
3 years	1.4% (1.1–1.9)	0.3% (0.1–0.9)	1.4% (0.9–2.3)	3.4% (2.3–5.0)
4 years	2.1% (1.6–2.7)	0.8% (0.4–1.6)	1.8% (1.2–2.9)	4.6% (3.2–6.6)
5 years	2.4% (1.9–3.1)	0.8% (0.4–1.6)	2.0% (1.3–3.1)	6.0% (4.3–8.5)
SNA event				
3 years	3.4% (2.8–4.1)	0.9% (0.5–1.6)	3.6% (2.7–4.9)	7.4% (5.7–9.5)
4 years	4.5% (3.8–5.4)	1.4% (0.9–2.4)	4.6% (3.5–6.1)	9.8% (7.7–12.5)
5 years	5.5% (4.7–6.5)	2.1% (1.3–3.2)	5.4% (4.1–7.1)	11.8% (9.3–14.9)
Mortality, AIDS, or SNA				
3 years	5.2% (4.4–6.0)	1.7% (1.1–2.6)	5.5% (4.3–7.0)	10.4% (8.3–12.9)
4 years	6.7% (5.8–7.7)	2.4% (1.6–3.5)	6.9% (5.5–8.7)	13.7% (11.1–16.7)
5 years	8.3% (7.3–9.5)	3.6% (2.6–5.0)	8.7% (7.0–10.8)	16.1% (13.3–19.5)

Note: Rates were estimated using the Kaplan-Meier method with log-log 95% confidence limits. Note: all log-rank p-values for a difference between age strata are <0.001 for all event types except AIDS (p = 0.049).

We examined determinants of the composite outcome of all-cause mortality, SNA, or AIDS in unadjusted and adjusted Cox models (Table 4). The following factors were considered individually in unadjusted models as well as together in a multivariable model: age, gender, injecting drug use, race, time since HIV diagnosis, years on cART, baseline and nadir CD4+ count, prior AIDS, prior CVD, diabetes mellitus status, body mass index, use of blood pressure lowering medication, use of lipid lowering medication, IL-6, and D-dimer. While several factors were associated with the composite in unadjusted analysis, the only risk factors that remained significant after adjustment for other covariates were age, male gender, use of antihypertensive drugs, and elevated levels of IL-6 and D-dimer (Table 4). A sensitivity analysis with use of the SMART cohort only where smoking status could be addressed confirmed that age, smoking, and elevated levels of IL-6 and D-dimer were significant risk factors for the composite endpoint, but the effects of male gender and antihypertensive medications were attenuated. A further sensitivity analysis adjusting for exposure to PIs, NNRTIs, abacavir, tenofovir, stavudine, zalciatabine, and lamivudine use, which were higher among older participants, did not attenuate risk estimates for older age, male gender, antihypertensive medications, or elevated biomarker levels.

**Table 4:** Unadjusted and multivariable risk prediction models of composite endpoint of all-cause mortality, AIDS, and SNA events.

Predictor	Unadjusted Models		Multivariable Model	
	HR (95% CI)	P-value	aHR (95% CI)	P-value
Age (per 10 years)	1.7 (1.5–2.0)	<0.001	1.4 (1.2–1.6)	<0.001
Years diagnosed (per 5 years)	1.3 (1.2–1.5)	<0.001	1.0 (0.9–1.2)	0.79
Years on cART (per 5 years)	1.6 (1.3–1.9)	<0.001	1.3 (1.0–1.8)	0.05
Female gender	0.6 (0.4–0.8)	0.002	0.6 (0.4–0.9)	0.007
Injecting drug user	1.6 (1.2–2.3)	0.006	1.4 (0.9–2.0)	0.10
Black race	1.1 (0.8–1.5)	0.63	1.0 (0.7–1.5)	0.86
Previous AIDS illness	1.2 (0.9–1.6)	0.14	0.9 (0.7–1.3)	0.64
IL-6* at baseline (per 1 SD increase)	1.5 (1.4–1.7)	<0.001	1.3 (1.2–1.5)	<0.001
D-dimer* at baseline (per 1 SD increase)	1.4 (1.3–1.6)	<0.001	1.3 (1.1–1.5)	<0.001
Body mass index (per 5 kg/m^2^) at baseline	1.1 (0.9–1.2)	0.40	0.9 (0.8–1.0)	0.46
Previous CVD at baseline	3.2 (1.8–5.4)	<0.001	1.4 (0.8–2.6)	0.27
Type-2 diabetes mellitus at baseline	2.0 (1.3–3.2)	0.003	1.2 (0.7–2.0)	0.48
On antihypertensive medication at baseline	2.7 (2.0–3.7)	<0.001	1.5 (1.0–2.2)	0.04
On antihyperlipidemia medication at baseline	1.9 (1.4–2.6)	<0.001	1.2 (0.8–1.6)	0.36
Baseline CD4^+^ cell count (per 100 cells/mm^3^)	1.0 (1.0–1.1)	0.82	1.0 (0.9–1.1)	0.76
Baseline nadir CD4^+^ cell count (per 100 cells/mm^3^)	1.0 (0.9–1.0)	0.38	1.0 (0.9–1.1)	0.92

Note: Multivariable model includes all covariates in the table. * Biomarkers are log-transformed and standardized to a mean of 0 and standard deviation of 1. A sensitivity analysis restricting to SMART participants to examine the effect of smoking status indicated that smoking significantly predicted mortality or non-AIDS morbidity unadjusted (HR 1.9, 95% CI 1.3–2.7) and adjusted for other covariates (aHR 1.9, 95% CI 1.2–2.9). Adjustment for smoking attenuated the adjusted risk associated with female gender (aHR 0.7, 95% CI 0.4–1.3) and antihypertensive medication use (aHR 1.5, 95% CI 0.9–2.4) but not for age (aHR 1.6, 95% CI 1.2–2.0), IL-6 (aHR 1.3, 95% CI 1.1–1.6) or D-dimer (aHR 1.3, 95% CI 1.1–1.6). Another sensitivity analysis adjusting for history of exposure to PIs, NNRTIs, abacavir, tenofovir, stavudine, zalcitabine, and lamivudine, which were significantly higher among older participants, did not notably alter the magnitude or significance of aHRs from the multivariable model above.

## Discussion

In SMART and ESPRIT, HIV-positive participants taking suppressive cART with high CD4^+^ cell counts were followed for several years and fatal and non-fatal AIDS, SNA and other causes of deaths were centrally adjudicated against standard event criteria. In this report we estimate rates of these major causes of morbidity and mortality overall and by age. Such information is critical for planning future research aimed at improving the long-term health among a growing number of older persons with HIV.

Over 90% of major clinical events among participants aged ≥50 years at enrollment were attributable to conditions other than AIDS, primarily CVD and non-AIDS cancers. This emphasizes the importance of prevention and control of established major CVD risk factors for an aging population with HIV [25], [26], and also motivates the study of interventions that address the underlying causes of the increased risk of SNA events such as anti-inflammatory treatments. To illustrate the utility of the event rate estimates in our report, we computed sample size assuming the target number of events needed was 380. For example, this event target might correspond to a placebo-controlled trial of a novel treatment to reduce inflammation for which 80% power to detect a 25% reduction in the hazard for the event of interest at the two-sided 0.05 significance level was assumed. Using the rates in Table 2 for those 50 years and older and assuming they are constant over a five-year follow-up period, we estimated the total number of patients required assuming patients were enrolled over a two-year period and followed for a minimum of three years. For the composite outcome of AIDS, SNA or death, the required sample size is 3,410; for fatal or non-fatal CVD, the required sample size is 9,260. Clearly, if an intervention is assumed to have a broader effect than on CVD, an assumption that is reasonable based on the relationship of biomarkers like IL-6 with different outcomes [23], [27]–[29], a composite outcome may be an efficient approach provided components of the composite can be assumed to move in line with one another in response to an intervention. This was the logic behind using such an outcome in the ongoing INSIGHT trial on the risks and benefits of early ART, the Strategic Timing of AntiRetorviral Therapy (START) trial [30]. Sample size would be larger if younger patients were enrolled; sample size would be smaller if a higher risk group (e.g., smokers or those with elevated D-dimer or IL-6) were enrolled. Recent ideas for risk stratification based on biomarkers and randomization to elements of a “polypill” [31], [32] may have merit for those at intermediate risk of CVD and are relevant for HIV patients. Sample size for treatments used with cART will also have to consider adherence and potential side effects of the randomly assigned treatments.

Consistent with previous studies [33], risk of grade 4 adverse events increased with older age across a number of body systems. This increased risk of toxicities with older age and increased use of other drugs besides cART, highlights the importance of identifying simple interventions for which adherence will be excellent over the long-term in order to reduce morbidity and mortality from serious non-AIDS conditions. It also indicates that toxicity management will become increasingly important as the age distribution of persons with HIV infection shifts older.

Strengths of the current study include the large geographically diverse population, long follow-up with little missing data, and use of standardized event definitions. The SMART and ESPRIT studies were among the earliest to carefully adjudicate non-AIDS conditions. A limitation is that participants were enrolled in clinical trials and may therefore be healthier than the general HIV population. However, our primary goal is to provide information useful for planning trials of future interventions, therefore data presented here are considered very relevant for that purpose. Another limitation is that the cohort largely consists of participants in developed countries and causes of morbidity and mortality for participants on suppressive cART with high CD4^+^ counts may be different in developing countries. An additional limitation is that older participants had different cART exposure than younger participants, both duration and type of drugs. However, in a sensitivity analysis, adjusting for these different cART exposures, duration of cART use, and other covariates did not change the associations of other covariates with the composite outcome.

In summary, the patterns of risk with age identified in this report highlight that major morbidity and mortality will be increasingly dominated by non-AIDS conditions and grade 4 events as individuals with HIV age. The age spectrum of persons with HIV is shifting older worldwide, largely as a consequence of effective treatment with cART. In the United States, the CDC estimates that half of all HIV-infected individuals will be aged 50 or older by 2015. Thus, event rates presented here will be useful for planning intervention studies aimed at interventions to treat or prevent non-AIDS diseases among persons with HIV taking suppressive cART.
